# Epigenetic Variation in Mangrove Plants Occurring in Contrasting Natural Environment

**DOI:** 10.1371/journal.pone.0010326

**Published:** 2010-04-26

**Authors:** Catarina Fonseca Lira-Medeiros, Christian Parisod, Ricardo Avancini Fernandes, Camila Souza Mata, Monica Aires Cardoso, Paulo Cavalcanti Gomes Ferreira

**Affiliations:** 1 Diretoria de Pesquisa Científica, Instituto de Pesquisas Jardim Botânico do Rio de Janeiro, Rio de Janeiro, Rio de Janeiro, Brasil; 2 Laboratoire de Biologie Cellulaire, Institut J.-P. Bourgin - INRA Centre de Versailles, Versailles, France; 3 Instituto de Bioquímica Médica, Universidade Federal do Rio de Janeiro, Rio de Janeiro, Rio de Janeiro, Brasil; University of Leeds, United Kingdom

## Abstract

**Background:**

Epigenetic modifications, such as cytosine methylation, are inherited in plant species and may occur in response to biotic or abiotic stress, affecting gene expression without changing genome sequence. *Laguncularia racemosa*, a mangrove species, occurs in naturally contrasting habitats where it is subjected daily to salinity and nutrient variations leading to morphological differences. This work aims at unraveling how CpG-methylation variation is distributed among individuals from two nearby habitats, at a riverside (RS) or near a salt marsh (SM), with different environmental pressures and how this variation is correlated with the observed morphological variation.

**Principal Findings:**

Significant differences were observed in morphological traits such as tree height, tree diameter, leaf width and leaf area between plants from RS and SM locations, resulting in smaller plants and smaller leaf size in SM plants. Methyl-Sensitive Amplified Polymorphism (MSAP) was used to assess genetic and epigenetic (CpG-methylation) variation in *L. racemosa* genomes from these populations. SM plants were hypomethylated (14.6% of loci had methylated samples) in comparison to RS (32.1% of loci had methylated samples). Within-population diversity was significantly greater for epigenetic than genetic data in both locations, but SM also had less epigenetic diversity than RS. Frequency-based (G_ST_) and multivariate (β_ST_) methods that estimate population structure showed significantly greater differentiation among locations for epigenetic than genetic data. Co-Inertia analysis, exploring jointly the genetic and epigenetic data, showed that individuals with similar genetic profiles presented divergent epigenetic profiles that were characteristic of the population in a particular environment, suggesting that CpG-methylation changes may be associated with environmental heterogeneity.

**Conclusions:**

In spite of significant morphological dissimilarities, individuals of *L. racemosa* from salt marsh and riverside presented little genetic but abundant DNA methylation differentiation, suggesting that epigenetic variation in natural plant populations has an important role in helping individuals to cope with different environments.

## Introduction

Epigenetic changes can modify phenotypes without changing nucleotide sequence of promoter or coding regions of a gene [1]. Covalent modifications of the DNA or histones are responsible for transmitting epigenetic information from cell to daughter cell and, in plants, from generation to generation [2], [3]. These phenomena are important to maintain genome stability against the proliferation of transposable elements, but also to control the regulation of gene expression [4]–[6]. CpG and non-CpG sequences are heavily methylated in the pericentromeric and repetitive regions of plant genomes, but DNA methylation is also present in gene-rich regions [7]. Accordingly, evidence that such DNA methylation is crucial for several plant developmental processes has accumulated in a short period [8]–[12]. The mechanisms controlling DNA methylation and their effects on phenotypes have been investigated in model plants [13], but their impact on the evolution of natural populations is underexplored [14], [15]. Unlike genetic variation, epigenetic changes generating novel and heritable phenotypes may represent reversible genomic alterations that allow colonizing variable environments and new landscapes on an evolutionary timescale [7], [16], [17]. Mangroves are ecosystems occurring along tropical and subtropical coastlines, and subjected to daily variations of water salinity due to sea level oscillations [18], [19]. Mangrove plant species thus have to tolerate a wide range of environmental conditions and often present divergent structural and morphological characteristics in different ecogeographic zones [20], [21]. In regions with abundant input of fresh water, nutrients and sediments, trees generally show good development and reach heights of over 40 meters [21]. In contrast, in habitats with limiting factors, such as periodic drought and hyper-saline soils (i.e. salt marshes), plants have abnormal development and reach heights of only 1.5 to 3 meters, with a shrub-like morphology [21].

**Figure 1:**
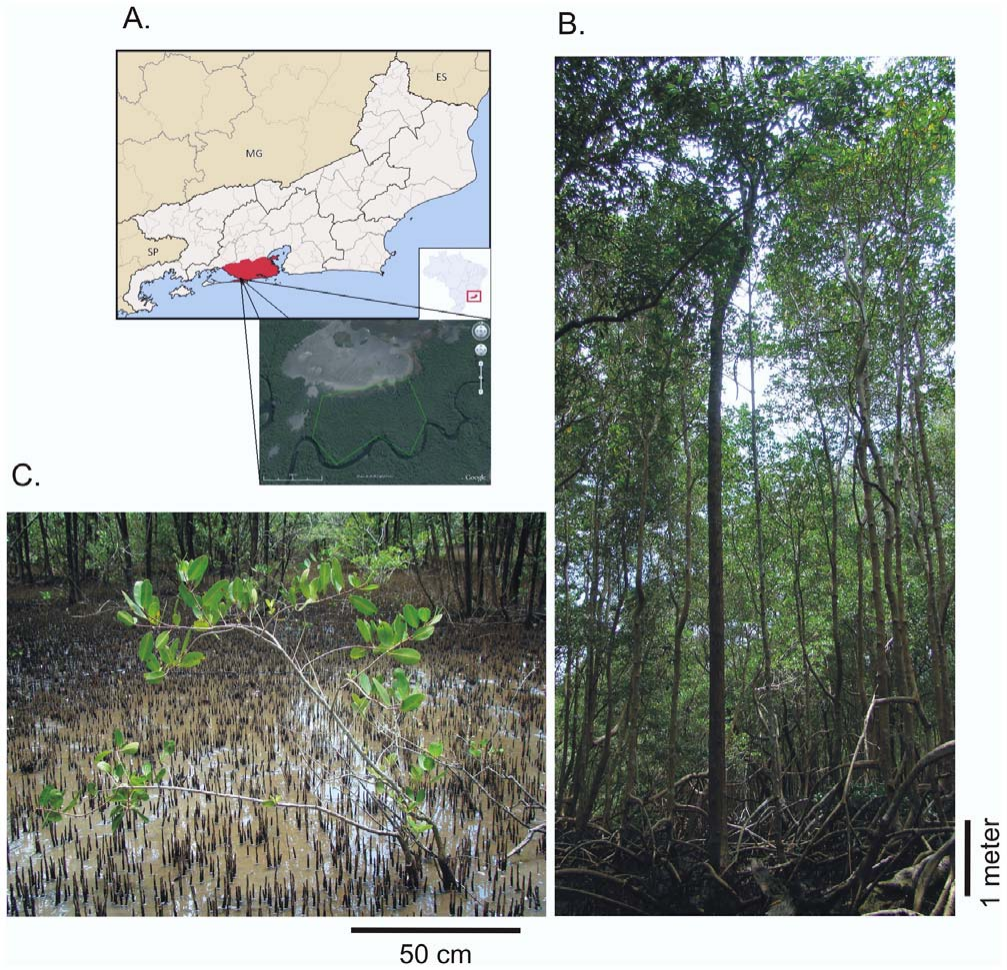
Map of the mangrove forest and pictures of *Laguncularia racemosa* illustrating the morphological differences between natural populations. (**A**) Map of Rio de Janeiro State, where the city of Rio de Janeiro is painted in red. There is an aerial view of the conserved Sepetiba Bay's mangrove forest. The salt marsh formation is visible in gray (no vegetation) above the study area delimited by a green line. (**B**) Individual of *L. racemosa* from the riverside (RS) location, almost 10 meters tall. (**C**) Typical *L. racemosa* individual from the area near a salt marsh (SM) with abnormal development, reaching only 1.5 meters in height.

*Laguncularia racemosa* (L.) Gaertn. f. (Family: Combretaceae), also known as white mangrove, is broadly found in the western world among mangroves of the Americas and Africa [20]. In Sepetiba Bay (Rio de Janeiro, Brazil; Figure 1A), individuals of *L. racemosa* are found either in a river basin or near a salt marsh, suggesting that plants located at each site might be under divergent environmental pressures. As postulated by Schaeffer-Novelli *et al.* [21], those individuals are morphologically distinct, having a tree-like structure in the riverside location (RS; Figure 1B) and a shrub-like morphology near the salt marsh (SM; Figure 1C). In a preliminary survey, samples from both RS and SM areas were characterized by Amplified Fragment Length Polymorphism (AFLP) markers and presented no significant genetic differentiation (C.F. Lira-Medeiros, unpublished data). Considering the remarkable genetic similarity, morphological differences between individuals from RS and SM areas are surprising and could be associated with epigenetic variation.

Few molecular tools are available to investigate epigenetic variation in non-model species, but the Methyl-Sensitive Amplified Polymorphism (MSAP) technique provides data on cytosine methylation without *a priori* knowledge of genome sequences [22]. Using two isoschizomer restriction enzymes (*Msp*I and *Hpa*II), which recognize the same restriction site (5-CCGG-3) but have different cytosine methylation sensitivities, MSAP allows the identification of CpG-methylation polymorphism [6], [23]–[25]. While several studies evaluated methylation changes among related lineages of crop and/or polyploidy species [9], [26]–[31], the potential of MSAP to analyze genetic and epigenetic structure of natural populations is still underexplored. Using MSAP, this study aims at describing how CpG-methylation is distributed among individuals from natural populations occurring in two contrasting habitats. It also explores correlations between environmental and morphological variations found in *L. racemosa*. This is one of the first reports to assess epigenetic variation in natural plant populations and it indicates that DNA methylation may be evolutionarily unlinked from genetic alterations to shape phenotypic variation.

## Results

### Morphological variation

Morphological traits such as tree height, tree diameter and leaf size were measured on 50 *Laguncularia racemosa* individuals from RS and SM populations in Sepetiba Bay's mangroves (Figure 1), half from each site. Tree heights in RS plants (mean 35.3 m) were significantly higher than in SM plants (mean 4.7 m) using two-sample t-test (t = 16.24, P<0.0001; Table 1). Tree diameters at breast height (DBH) were also significantly different with mean values of 35.3 cm and 4.7 cm for RS and SM plants respectively (t = 14.26, P<0.0001; Table 1).

**Table 1:** Morphological data obtained from *Laguncularia racemosa* plants in Sepetiba Bay's mangrove forest.

	H (m)	DBH (cm)	L (cm)	W (cm)	A (cm^2^)
**RS**	7.5***	35.3***	7.3^NS^	4.5***	25.3***
**SM**	1.9***	4.7***	7.2^NS^	4.0***	23***

*** P<0.0001.

NS non-significant.

Mean of tree height (H), diameter at breast height (DBH), leaf length (L), leaf width (W) and leaf area (A) were calculated for *L. racemosa* plants in RS and SM areas. Welch Two Sample t-test was performed to obtain P values.

Variation in leaf morphology were assessed by measuring leaf length, leaf width and leaf area in 25 leaves of each tree analyzed above (N = 1250; Table 1). Leaf length did not deviate significantly between RS and SM samples, with means of 7.3 cm and 7.2 cm respectively (t = 1.83, P = 0.068). In contrast, leaf width was significantly different between the two locations with means of 4.5 cm in RS and 4.0 cm in SM (t = 7.23, P<0.0001). Leaf areas were also significantly different between RS and SM plants with means of 25.3 cm2 and 23 cm2, respectively (t = 4.92, P<0.0001).

### Methylation patterns

Six primer-pair combinations provided 209 reliable MSAP loci to study 34 *L. racemosa* individuals, half from each site (RS and SM). The calculated error rate was approximately 3% based on negative controls and replicated samples. Number of loci counted for each primer combination varied from 10 to 70 (Table 2). Comparisons between *Eco*RI/*Msp*I and *Eco*RI/*Hpa*II profiles for each sample allowed us to identify non-methylated, methylated and hemimethylated loci based on methylation sensitivities of both isoschizomers [32]. Presence of MSAP fragments in both *Eco*RI/*Msp*I and *Eco*RI/*Hpa*II profiles indicated non-methylated loci. CpG-methylated loci were characterized by the presence of *Eco*RI/*Msp*I fragments and absence of *Eco*RI/*Hpa*II fragments in the same locus [32], [33]. The opposite pattern, fragments present in *Eco*RI/*Hpa*II digestions but absent in *Eco*RI/*Msp*I digestions, were counted as hemimethylated loci representing methylation on external cytosines (i.e. non-CpG methylation [32]).

**Table 2:** Number of loci and methylation pattern analyzed per *Eco*RI and *Msp*I/*Hpa*II primer combination.

*Eco*RI selective primer	*Msp*I/*Hpa*II selective primer	Methylated *Loci*	Non-methylated *Loci*	Hemi methylated *Loci*	*Loci* per primer combination
AG	TCAA	14	12	4	30
AAC	TCAA	3	7	0	10
AC	TCAA	5	15	3	23
AT	AAT	16	17	9	42
AG	AAT	9	23	2	34
AAC	AAT	20	42	8	70
	**Total**	**67 (32.1%)**	**116(55.5%)**	**26 (12.4%)**	**209**

Number of methylated, non-methylated and hemimethylated loci found with each of the six *Eco*RI and *Msp*I/*Hpa*II primer combinations used for *L. racemosa* population genetic analyses. A total of 209 loci were found from which 183 were used on the genetic and epigenetic analyses, after excluding hemimethylated loci (details in Methods).

Considering all MSAP markers, we observed 67 loci with CpG-methylation, 116 non-methylated loci and 26 hemimethylated loci (Table 2). All 67 methylated loci were found methylated in some samples of RS population (32.1% of all loci) but only 30 of these loci were methylated in some samples of SM population (14.6% of all loci). In this case, the remaining 37 loci were not methylated in any sample, indicating a hypomethylation in the SM population. The methylation status of all methylated loci also varied between samples from the same population. We observed that only 13 loci out of the 67 methylated loci were methylated in all RS samples. And all SM samples were methylated at the same time in 18 loci out of 30 methylated loci, indicating less CpG-methylation variation in SM plants.

### Genetic structure

The genetic structure of *L. racemosa* was analyzed using *Eco*RI/*Msp*I data of 183 MSAP loci. Hemimethylated loci (i.e. loci with fragments present in *Eco*RI/*Hpa*II digestions but absent in *Eco*RI/*Msp*I digestions) were excluded from genetic and epigenetic analysis. This type of fragment is not inherited over generations and represented only 12.4% of all loci obtained in the present study (Table 2).

**Figure 2:**
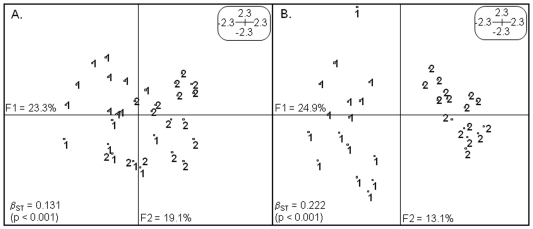
PCA analyses of *Laguncularia racemosa* natural populations using both genetic and epigenetic data separately. Multivariate analysis of the genetic (*Eco*RI/*Msp*I) and epigenetic (*Eco*RI/*Hpa*II) components of *L. racemosa* plants found at riverside (RS represented as number 1) and the salt marsh (SM represented as number 2) locations of Sepetiba's Bay mangrove forest. (**A**) PCA on covariance matrix for genetic profiles obtained using *Eco*RI/*Msp*I data. (**B**) PCA on covariance matrix for epigenetic profiles obtained using *Eco*RI/*Hpa*II data. F1 and F2 values show the contribution of the two principal components summarizing the total variance of each dataset. β_ST_ was calculated using Between-group EigenAnalysis (BPCA) for both genetic and epigenetic profiles and tested with 9999 permutations.

Within-population Shannon diversity indices for genetic data were 0.013 and 0.008 for RS and SM, respectively, and not significantly different (Wilcoxon rank sum test; W = 16031.5, P = 0.06; Table 3). The Principal Component Analysis (PCA) based on covariance matrix summarized 42.4% of the total inertia in the two first principal components of *Eco*RI/*Msp*I data (Figure 2A), and showed very small genetic differences between RS and SM populations, with individuals from different locations being genetically very similar (i.e. central samples on Figure 2A).

**Table 3:** Within-population diversity and among-population differentiation of genetic and epigenetic components of *Laguncularia racemosa* genome.

Data	Location	Shannon diversity	G_ST_	β_ST_
*Eco*RI/*Msp*I	RS	0.013^a^	0.152	0.131***
	SM	0.008^a^		
*Eco*RI/*Hpa*II	RS	0.084^b^	0.183	0.222***
	SM	0.024^c^		

*** P<0.0001.

Within-population Shannon diversity index was calculated for each dataset and each *L. racemosa* population. Differentiation indices between populations were calculated using three statistical methods. *Eco*RI/*Msp*I data represent genetic profiles while *Eco*RI/*Hpa*II data represent epigenetic profiles. Values with different superscript letters are significantly different according to Wilcoxon rank sum test with continuity correction and Kruskal-Wallis rank sum test.

Since dominant markers cannot be used to calculate deviation from the Hardy-Weinberg equilibrium directly, two complementary statistical methods, which do not rely on these assumptions, were used and showed similar values for *Eco*RI/*Msp*I data. Based on the partition of Shannon diversity within and among populations, we calculated a G_ST_ of 0.152 (Table 3). The multivariate Between-group Eigen Analysis (BPCA – PCA among groups based on PCA among individuals) resulted in a significant β_ST_ of 0.131 (P<0.001; Table 3).

### Epigenetic structure

The same 183 loci were similarly investigated for epigenetic structure of *L. racemosa* using *Eco*RI/*Hpa*II dataset. The within-population epigenetic Shannon indices calculated for RS and SM were 0.084 and 0.024, respectively, and were significantly different (Wilcoxon rank sum test; W = 11459.5, P<0.001; Table 3). Comparisons between genetic and epigenetic Shannon indices of the same population resulted in significantly higher epigenetic values in both locations (Kruskal-Wallis chi-squared = 112.8326, P<0.0001 for RS; and Kruskal-Wallis chi-squared = 143.1622, P<0.0001 for SM). The PCA based on covariance matrix of *Eco*RI/*Hpa*II data summarized 38% of the total inertia in the two first principal components and showed pronounced differentiation between RS and SM samples. Individuals from different locations were clearly separated, and SM plants were more similar to each other than RS individuals on the epigenetic profile (Figure 2B).The epigenetic structure was also evaluated using two statistical methods, which gave similar differentiation indices: G_ST_ = 0.183 and β_ST_ = 0.222 (P<0.001; Table 3). Frequency-based and multivariate methods showed greater epigenetic than genetic differentiation between *L. racemosa* natural populations, as can also be visualized on PCAs (Figure 2).

### Genetic *versus* epigenetic structure

In order to evaluate the contribution of both genetic and epigenetic profiles to the *L. racemosa* population structure, Co-Inertia analysis was performed. This analysis is a multivariate method that maximizes shared structure among multiple datasets drawn from the same samples. The two first axis of Co-Inertia analysis explained 76.5% of the genetic co-variation between *Eco*RI/*Msp*I and *Eco*RI/*Hpa*II datasets and this association was significantly different from the value expected for random association (P<0.0001).

**Figure 3:**
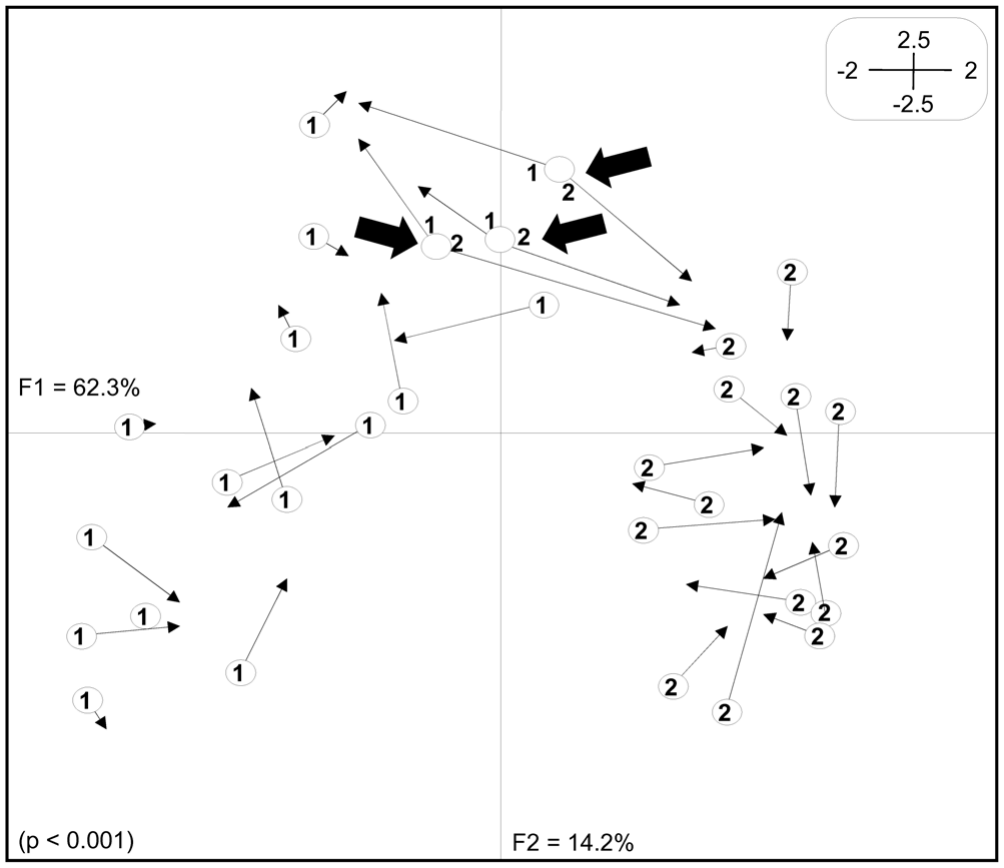
Co-Inertia analysis of *Laguncularia racemosa* natural populations using genetic and epigenetic data. The Co-Inertia analysis maximized the covariance of PCAs shown in Figure 2. The significance test of this association was done with 9999 permutations. RS samples are numbered as 1 while SM samples are numbered as 2. Circles (○) correspond to the projection of genetic profiles (*Eco*RI/*Msp*I) and arrowheads (▸) the projection of epigenetic profiles (*Eco*RI/*Hpa*II). Black-filled arrows indicate three RS samples that had similar genetic profiles with three SM samples, but divergent epigenetic profiles. F1 and F2 values show the contribution of the two principal components summarizing the total variance of each dataset.

In the Co-Inertia subspace (Figure 3), three individuals from the RS population (represented by 1) and three from the SM population (represented by 2) showed similar genetic profiles (represented as circles: ○), but rather divergent epigenetic profiles (represented as arrowheads: ▸). The epigenetic profiles of these samples point in opposite directions, where RS samples clearly have epigenetic profiles (▸) more similar to other plants from RS than to plants from the SM population. The same is true for SM samples, which are more similar to their co-habitants than to RS plants. Co-Inertia also showed less variation in both genetic and epigenetic components in SM than in RS samples. This is represented by a greater aggregation of SM circles (genetic profiles) and arrowheads (epigenetic profiles) in the Co-Inertia graph (Figure 3).

## Discussion

Mangrove zonation is affected by climatic and hydrologic factors that shape the structural development of mangrove stands [21], [34]. In Sepetiba Bay, mangrove zonation results in two physiographic forests where *L. racemosa* individuals display very distinct morphological characteristics. Riverside plants (RS) undergo daily tidal fluxes and consequently high nutrient input whereas salt marsh plants (SM) suffer from high soil salinity and low nutrient input; consequently plants from these areas develop differently.

Our analyses of morphological traits in *L. racemosa* plants from RS and SM area correlate morphology with mangrove zonation. Tree height and trunk diameter reflected tree-like structure of RS plants and shrub-like structure of SM plants. Mangrove forests near salt marshes have been classified as dwarf forests [34], because they have abnormal growth (Figure 1). The leaf measures also showed significant reduction of leaf width and leaf area in SM plants that can be associated with environmental restrictions of their habitat. Another mangrove species *Rhizophora mangle* was also reported to have smaller leaves as a result of salinity and nutrient deficiency in salt marsh areas [35].

Preliminary analysis using AFLP markers revealed very little genetic differentiation (G_ST_ = 0.032) between *L. racemosa* individuals from SM and RS sites (C.F. Lira-Medeiros, unpublished data), suggesting that the extensive morphological divergence between the two sites was most affected by epigenetic events.

Using MSAP methodology, we assessed both genetic (*Eco*RI/*Msp*I) and epigenetic (*Eco*RI/*Hpa*II) components of the genome of *L. racemosa* plants from RS and SM areas. The use of these isoschizomers concentrates our investigation on CpG-dinucleotide regions, which are generally gene-rich areas of the genome [36]. Moreover, after the exclusion of hemimethylated loci, we could bypass *Msp*I sensitivity to non-symmetrical methylated sites and extrapolate the *Eco*RI/*Msp*I data analysis as genetic profile. Thus MSAP markers were highly informative, revealing genetic and epigenetic components of *L. racemosa* genome in natural populations.

Divergences in CpG-methylation levels were detected by comparing *Msp*I and *Hpa*II profiles at the same loci. In plants from the RS location, 32.1% of all MSAP fragments were methylated in some samples, while only 14.6% of loci from SM plants were methylated in some samples. Previous works have shown that 30–41% of loci were methylated in *Brassica oleraceae* accessions [32], 32% of loci were methylated in *Gossypium hirsutum* accessions [29], and 35–43% of loci were methylated in *Arabidopsis thaliana* ecotypes [23]. This indicates a hypomethylation in the genome of SM plants. The hypomethylation has already been shown to affect plant development in other studies [37], [38].

Genetic profiles observed in this work showed some similarities between RS and SM samples, but epigenetic profiles were more differentiated as shown by PCAs (Figure 2) and differentiation indices (Table 3).

Two statistical methods resulted in higher epigenetic than genetic differentiation, suggesting that the two components of the genome probably are under divergent evolutionary pressures resulting in divergent genetic and epigenetic structure of RS and SM samples. At the epigenetic level, the Shannon index was significantly greater within the RS than within the SM population, showing that epigenetic diversity is somewhat constrained in the SM habitat. It is the first time that natural epigenetic variation is shown in a wild plant population and it is unexpected to learn that genetic and epigenetic diversity is not always linked together. Since the RS population is located in more favorable habitat of mangrove forest with daily tides and high nutrient inputs, and SM plants are subjected to limited nutrient input and high saline soil, we believe that it is expected that the population under stress would have changes in their epigenome in order to cope with habitat conditions. The outcome is an increased number of both fixed methylated loci and non-methylated loci resulting in the erosion of the epigenetic diversity in the SM population and an abnormal development of the plants.

Co-Inertia analysis (Figure 3) also showed greater epigenetic than genetic variation between RS and SM plants. Interestingly, similarities between individuals from the same population are stronger at the epigenetic than at the genetic level, indicating that environmental conditions might have shaped epigenetic differentiation between the two locations. Apparently, the genetic component of the genome is not strongly affected in the same way by the habitat. Similar results were found in *Solanum* natural populations where epigenetic variation was greater than genetic variation and associated with abnormal floral phenotypes [39]. MET1 is a plant DNA methyltransferase that plays an important role in CpG-dinucleotide methylation maintenance on single-copy and repeat DNA [40]. Hypomethylated genomes caused by MET1 deficiency has been associated with development abnormalities such as plant stature, reduced apical dominance and decreased fertility [41]. Active methylation and demethylation of DNA is a key factor for sensing environmental changes and reacting with change in gene expression, especially in plants [42].

*Laguncularia racemosa* populations are interesting natural systems to study the correlations between DNA methylation level, environmental conditions and morphological traits. It is attractive to postulate an association between hypomethylated plants near the salt marsh and their unique developmental pattern by demethylation in these stressed plants. DNA methylation patterns are maintained by MET1 gene, which may be responsible for the methylation variation found in *L. racemosa* populations. A reduced level of this enzyme could lead to hypomethylation of SM plants. Further investigations should be carried on to confirm this hypothesis.

We suggest that the epigenetic component of a genome, currently underexplored, plays an important role in long-term adaptation of the species in different environmental conditions. Epigenetic markers should not be disregarded in future population studies.

## Materials and Methods

### Plant material

*Laguncularia racemosa* plants in Sepetiba Bay's mangrove forest (Rio de Janeiro, Brazil; Figure 1A) located within a 16-ha area limited by a salt marsh on one side and the Piraco River on the other side were investigated. Individuals near the salt marsh (SM) or at the riverside (RS) are 200 meters apart, separated by a transitional area called *Rhizophora* Sea where only the mangrove species *Rhizophora mangle* occurs. Young and undamaged *L. racemosa* leaves from 17 randomly chosen adult trees from each location were sampled and immediately stored in silica gel for DNA analyses.

### Morphological analyses

Morphological traits were measured on 50 randomly-chosen *L. racemosa* individuals in the two locations of Sepetiba Bay's mangrove using methodology described elsewhere [43]. We measured tree height and diameter at breast height (DBH) of 25 plants from each location, RS and SM. The results were tested for significant differences between sites using the Welch Two Sample t-test in R software [44]. We also collected 25 leaves from the distal end of upper branches of those 50 measured trees in order to measure leaf length (L), leaf width (W) and leaf area (A). The leaf area (cm2) was calculated using a formula developed for *L. racemosa* plants by K.W. Krauss (unpublished data). These three leaf traits were tested for significant divergence between RS and SM plants using Welch Two Sample t-test in R software [44].

### Genetic/Epigenetic analyses

DNA extraction was carried out based on the protocol described by Cardoso *et al.* [45] with some modifications, including a scale-down process using 50 mg of dry material. PVP 40 000 was used to improve DNA yield and quality. DNA was resuspended in 100µl of sterile water and quantified on 1% agarose gels.

MSAP methodology used both *Eco*RI/*Msp*I and *Eco*RI/*Hpa*II digests as described by Xiong *et al.* [22]. Samples were subjected to *Eco*RI digestion using 1µg of genomic DNA and 10 U of enzyme (Promega^R^) with 1× buffer H in a final volume of 200µl. Digested DNA was precipitated with 0.1 vol of 3 M sodium acetate and 2.5 vol of 100% ethanol and afterwards washed in 70% ethanol. Half of *Eco*RI-digested DNA was used for digestion by each isoschizomer enzyme, with 5 U of *Msp*I in 1× Multicore buffer (Promega^R^) or 5 U of *Hpa*II in 1× Buffer B (Promega^R^) in a final volume of 50µl. Incubations were all performed at 37°C for 6 h and enzymes were afterwards denatured at 65°C for 20 min. Adapter ligation was performed with 20µl of digested DNA, 1× T4 DNA ligase buffer (Promega^R^), 1 U T4 DNA ligase enzyme (Promega^R^), 5 pmol of each *Eco*RI adapter [46] and 50 pmol of each *Msp*I/*Hpa*II adapter [22] in a 30µl reaction for 3 h at 20°C.

Pre-amplification was conducted in a 20µl reaction using 2µl of ligated and digested DNA, 1× PCR buffer, 0.4 mM dNTPs, 30 ng of *Eco*RI (5′-GACTGCGTACCAATTC-3′) and *Msp*I/*Hpa*II basic primers (5′-ATCATGAGTCCTGCTCGG-3′) and 2 U Taq polymerase (Ludwig Biotecnologia Ltda). The reactions were carried out for 25 cycles of 94°C 1 min, 56°C 1 min and 72°C 2 min with a 10-min final extension. Pre-amplification products were diluted 20 fold and 5µl was used for the selective amplifications. These amplifications use the basic primer sequence with 2 to 3 selective nucleotides at the 3′ end in order to obtain greater polymorphism with the same DNA digestions and to have at the same time DNA fragments that could be visualized in the gel. These 20µl reactions contained 30 ng of each selective primer *Eco*RI and *Msp*I/*Hpa*II (*Eco*RI+AG, *Eco*RI+AC and *Eco*RI+AAC combined with *Msp*I/*Hpa*II+TCAA and also *Eco*RI+AG, *Eco*RI+AAC and *Eco*RI+AT combined with *Msp*I/*Hpa*II+AAT), 0.2 mM dNTPs, 1× PCR buffer and 2 U Taq polymerase (Ludwig Biotecnologia Ltd). The touchdown program performed was: 94°C for 30 s, 65°C for 30 s and 72°C for 1 min decreasing the annealing temperature by 0.7°C per cycle during 12 cycles and then 24 cycles of 94°C for 30 s, 56°C for 1 min and 72°C for 2 min with a final period of 5 min at 72°C. The final amplification products were separated by electrophoresis for 2.5 h at 60 W on a 4% denaturing polyacrylamide gel with 7.5 M urea. Gels were stained with a 0.1% silver nitrate solution containing 0.5% formaldehyde for 30 min after gel fixation on 10% acetic acid solution for 20 min. Staining development used a 6% sodium carbonate solution with 0.5% formaldehyde and 2µg of sodium thiosulphate for 3 min and the reaction was stopped with 10% acetic acid solution. Silver-stained gels were photo-documented for manual scoring.

### Data analysis

The 34 samples were scored for presence (1) or absence (0) of *Eco*RI/*Msp*I and *Eco*RI/*Hpa*II fragments. Only unambiguously and intensely labeled bands were scored. The error rate (3%) was calculated based on negative controls and repeated samples [47]. Loci with different band pattern on controls or replications were excluded from analysis as possible methodological artifacts and recorded as errors. Three types of DNA methylation status were identified by presence (1) or absence (0) of *Eco*RI/*Msp*I and *Eco*RI/*Hpa*II digests respectively: fully methylated loci (1/0), non-methylated loci (0/0) and hemimethylated loci (0/1) as stated by Salmon *et al.* [32]. Hemimethylated loci are represented by methylation in one DNA strand but not in its complement strand, i.e. the external cytosine residue of *Msp*I and *Hpa*II restriction site (5-CCGG-3) is methylated in one DNA strand only. Since this type of fragment is not inherited over generations, they were excluded from population structure analyses of *L. racemosa*. These analyses were done using both *Eco*RI/*Msp*I and *Eco*RI/*Hpa*II profiles separately in order to obtain the genetic (represented by non-symmetrical methylation sensitivity of *Msp*I enzyme) versus epigenetic structure (represented by CpG-methylation sensitivity of *Hpa*II).

Dealing with dominant markers, heterozygosity cannot be calculated directly, so deviation from Hardy-Weinberg equilibrium thus has to be either: (i) assumed as null, (ii) bypassed, or (iii) assessed by other means [48], [49]. Here, the first two possibilities have been explored and compared to provide robust estimates of the genetic and epigenetic structure of *L. racemosa* populations using MSAP data of *Eco*RI/*Msp*I and *Eco*RI/*Hpa*II respectively.

First, within-population genetic diversity (H_pop_) was assessed by Shannon diversity index calculated based on the frequency of each band out of the 17 samples for *Eco*RI/*Msp*I and *Eco*RI/*Hpa*II. As recommended by Bussell [50], log2 (0) was replaced by 0 for fixed absent bands. Significant differences of Shannon index among populations for each genetic and epigenetic profiles were assessed using the Wilcoxon rank sum test with continuity correction and significant differences between genetic and epigenetic Shannon indices inside each population was calculated by Kruskal-Wallis rank sum test both using R software [44]. The significance of these tests was adjusted with the Bonferroni correction [51]. Genetic and epigenetic structures were computed for each *Eco*RI/*Msp*I and *Eco*RI/*Hpa*II profiles as *G*_ST_ = (H_tot_−H_pop_)/H_tot_ [50].

Second, individual profiles were also investigated by multivariate analyses because it is a band-based approach that does not assume Hardy-Weinberg equilibrium. Principal Component Analysis (PCA) on inter-profile covariance matrix followed by Between-group Eigen Analysis (BPCA [49]) was computed on *Eco*RI/*Msp*I and *Eco*RI/*Hpa*II data using ADE-4 [52]. BPCA (i.e. PCA among groups based on the PCA among individuals) divides the variance into within- and between-group components and, given that it is a Euclidean approach, can be considered as analogous to *F*-statistics (called here *β*_ST_). The statistical significance was assessed by the Romesburg randomization test (9999 permutations). In addition, multivariate analyses allow for the joint analysis of CpG-genetic and epigenetic structure through statistical procedures that maximize and test the common variance of different datasets. Here, the symmetrical Co-Inertia Analysis was used to investigate the association between *Eco*RI/*Msp*I and *Eco*RI/*Hpa*II profiles by projecting the PCA scores of individuals into a new subspace, maximizing their covariance [53]. Unlike the related Canonical Correspondence Analysis, Co-Inertia analysis does not rely on linear regressions and thus can be safely used for any number of variables to be related [54]. The significance of this association has been tested in ADE-4 by a procedure in which rows of *Eco*RI/*Msp*I and *Eco*RI/*Hpa*II tables were randomly permuted 9999 times.
